# Elevated Trans-mitral Pressure Gradient Predicts Surgery in Young People with Moderate–Severe Rheumatic Mitral Regurgitation

**DOI:** 10.1007/s00246-024-03660-0

**Published:** 2024-10-15

**Authors:** Jacqueline M. Williamson, Gillian A. Whalley, Ari E. Horton, Peter Morris, Bo Remenyi

**Affiliations:** 1 Child and Maternal Health Division, Menzies School of Health Research, Darwin, Australia; 2 Charles Darwin University, Darwin, Australia; 3 Department of Medicine, Otago School of Medicine, Otago University, Dunedin, New Zealand; 4 Victorian Heart Institute, Melbourne, Australia; 5 Monash Children’s Hospital, Monash Health, Melbourne, Australia; 6 Royal Darwin Hospital, Darwin, Australia; 7 Royal Darwin Hospital, 105 Rocklands Dr, Tiwi, Northern Territory 0810 Australia

**Keywords:** Trans-mitral pressure gradient, Mitral regurgitation, Echocardiography, Rheumatic heart disease, Mitral valve surgery

## Abstract

Mitral regurgitation (MR) is the most common lesion in children with rheumatic heart disease (RHD). Progression of RHD results in the need for surgical intervention, the timing of which is dictated by left ventricular dilatation and the onset of heart failure symptoms. We sought to determine whether elevation in trans-mitral pressure gradient (TMPG) in those with moderate or severe rheumatic MR without significant mitral stenosis (MS) could predict the need for future surgical intervention. Echocardiographic studies were reviewed for 116 children and young people with moderate or severe rheumatic MR. Those with significant mitral stenosis or concurrent aortic valve disease were excluded. Trans-mitral pressure gradient was measured at baseline and details of mitral valve surgical intervention were retrieved from a registry database. Time to future surgery (up to six years) was compared between those with TMPG < 5 mmHg and TMPG ≥ 5 mmHg. Survival curves demonstrated an increased risk of surgery for those with TMPG ≥ 5 mmHg with Cox proportional regression analysis providing a hazard ratio of 5.8. The proportion free from mitral valve surgery at one year for the TMPG < 5 mmHg group was 0.94 (95% CI 0.86–0.97), compared to 0.62 (95% CI 0.42–0.77) in the ≥ 5 mmHg group. Trans-mitral pressure gradient is a strong predictor of future mitral valve surgery in children and young people with significant rheumatic MR without MS. This non-invasive measure could be used to signal the need for more aggressive monitoring in order to optimize the timing of intervention.

## Introduction

The incidence of mitral regurgitation (MR) is high in children with rheumatic heart disease (RHD) and implicated in up to 97% of valvular lesions in those aged 20 years or younger [1]. Significant MR (moderate or severe) leads to left atrial (LA) and left ventricular (LV) dilatation, pulmonary hypertension, and eventually heart failure. Symptomatic relief may be achieved via medical therapy, including diuretics and angiotensin-converting enzyme inhibitors; however, surgical intervention may be required to repair or replace the mitral valve in those who remain symptomatic.

Echocardiography is the gold standard for the assessment and diagnosis of MR severity and its complications. Timing of surgery can be difficult to predict with some patients tolerating chronic severe MR for many months or years. Significant MR results in an increase in diastolic trans-mitral mean pressure gradient (TMPG) which can complicate the diagnosis of mitral stenosis (MS) where the two lesions co-exist [2]. An elevated gradient is typically associated with the presence of MS, and this gradient together with the mitral valve area (MVA) are used in the diagnosis of MS [3]. Trans-mitral pressure gradient is a measure of the pressure difference between the LA and LV during diastole and is highly dependent on loading. Normal TMPG in children of all ages is reported to be in the vicinity of 1 mmHg [4] with gradients in excess of 4 mmHg considered to be consistent with MS [5]. Conversely, in the setting of MR, clinicians largely ignore gradient and favor parameters, such as regurgitant volume, regurgitant fraction, and the secondary complications of LA dilatation and LV volume overload. Timing of surgical intervention is based on the presence of volume overload reflected in left ventricular dilatation and the development of heart failure symptoms [6].

To our knowledge, there is no published data evaluating the prognostic role of TMPG in the setting of isolated MR without significant MS in unrepaired native valves in adults or children. Noting this, we hypothesize that in children and young people with moderate or severe MR, elevated TMPG may be a predictor of worse outcome despite the absence of significant MS. The purpose of this investigation was to evaluate the prognostic value of elevated TMPG in children and young adults with moderate or severe rheumatic MR.

## Materials and Methods

### Study Population

The Northern Territory of Australia RHD Register, [a Department of Health record for compulsory notification of RHD and acute rheumatic fever (ARF)], was used to identify children and young adults (age ≤ 20 years) diagnosed with RHD between January 2012 and December 2021. Patients were included in this study if found to have moderate or severe mitral regurgitation, but no significant mitral stenosis during the period of follow-up which spanned to December 31, 2022. Of the 432 diagnosed with RHD, 140 children and young adults met the inclusion criteria. Those with suboptimal echocardiographic images, significant co-existing aortic valve disease, or mitral stenosis were excluded leaving a study population (*n*) of 116. Demographic data were captured along with echocardiographic parameters used to assess chamber size and evaluate mitral valve morphology and function. Images were reviewed on a server (Synapse, Fujifilm, Japan) with study data collected and managed using REDCap electronic data capture tool hosted at Menzies School of Health Research and Excel (Microsoft Corporation, 2018). Data regarding valvular surgical procedures were extracted from the Northern Territory RHD register.

Human research ethics approval was sought from Menzies School of Health Research with approval granted for this low-risk review of retrospectively captured clinical data (HREC: *2021–4146)*.

#### Echocardiographic Techniques

Echocardiograms were performed by accredited cardiac sonographers or pediatric cardiologists using E95, S70, Vivid I or Vivid Q ultrasound devices (GE Healthcare, USA). Post hoc echocardiographic measurements were performed offline by a single operator, (JW) using Synapse (Fujifilm, Japan) and compared to the original diagnostic data. Where values differed from the original report, measurements were repeated by a third party (GW) with final values decided by consensus agreement.

#### Mitral Regurgitation

Rheumatic mitral regurgitation is often posteriorly directed due to morphological changes of the mitral valve making quantitative assessment particularly difficult. The severity of MR was assessed through qualitative and semi-quantitative means, including subjective assessment of PISA formation, vena contracta width, spectral Doppler intensity, mitral inflow E wave velocity, and the presence/absence of pulmonary venous systolic flow reversal [7]. All patients included in this study had moderate or severe mitral regurgitation based on the characteristics described.

#### Mitral Valve Data

**Fig. 1:**
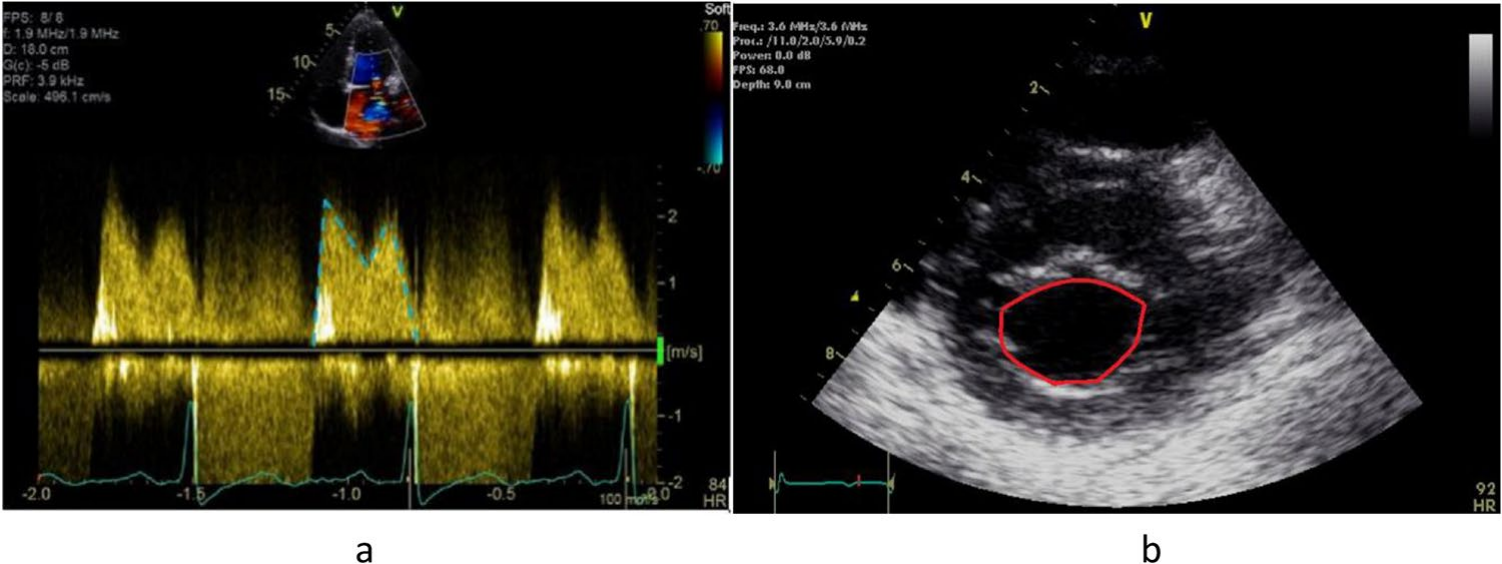
**a** Mitral inflow Doppler. Continuous wave Doppler measurement of TMPG (blue/green-dashed line) from the apical 4-chamber view. **b** Technique for estimating mitral valve area. Two-dimensional planimetry (red circular line) from a parasternal short-axis view was performed during diastole

An apical four-chamber view of the mitral valve was used to obtain TMPG with the modal velocity of the continuous wave Doppler signal traced after optimizing the image using post-processing techniques (Fig. 1a). Trans-mitral pressure gradient of < 5 mmHg is used to exclude the presence of significant mitral stenosis^3^ and this value was selected to stratify our group into low TMPG (< 5 mmHg) and elevated TMPG (≥ 5 mmHg) groups.

The presence of significant mitral regurgitation (which was often eccentric) determined that planimetry was the most appropriate method for obtaining MVA in this cohort [3]. Two-dimensional planimetry was performed from the parasternal short-axis view at the level of the mitral valve leaflet tips with the largest area traced during diastole (Fig. 1b). Three-dimensional echocardiographic imaging is considered the gold standard for planimetric determination of MVA [8]; however, this was not available in the remote communities where the majority of our studies were performed.

#### Left Atrial Size

Dilatation of the left atrium is common in the setting of chronic MR. Due to the limited nature of many of the echocardiographic studies which were performed in busy clinics in remote communities, left atrial size is estimated from a single-plane parasternal long-axis view. In order to index the atrial dimension to patient size, the aortic root (Ao) diameter was used as a comparison to determine the LA/Ao ratio. Aortic root dimension remains relatively unaffected by volume overload associated with MR and based on previous data, a cut-off of 1.5 was used to indicate the presence of left atrial dilatation [9].

The LA and aortic root dimension were both measured from a parasternal long-axis view using the leading edge to leading edge technique for M-mode measurements and inner edge to inner edge for two-dimensional measurements as recommended by the American Society of Echocardiography guidelines [10]. LA/Ao ratio was calculated by dividing the LA dimension by the Ao root dimension.

#### Left Ventricular Dimensions and Fractional Shortening

Left ventricular internal end-diastolic (LVIDd) and end-systolic (LVIDs) dimensions were measured from a parasternal long-axis view using the leading edge to leading edge technique for M-mode measurements and inner edge to inner edge for two-dimensional measurements as recommended by the American Society of Echocardiography guidelines [10]. The fractional shortening (FS%) was calculated by dividing the difference between the diastolic dimension and systolic LV dimension by the diastolic dimension ((LVIDd-LVIDs/LVIDd)*100). Fractional shortening of ≥ 26% is considered normal in children [11]; however, LV systolic function is often flattered by the presence of MR and the normal range should therefore be greater than this. An estimated LVEF of around 70% is considered normal in adults with significant MR [12] which equates to FS of 40%.

### Statistical Analysis

Stata V.14 (Stata Corp, Texas, USA) was used to perform survival analysis curves and all analyses. Mean and standard deviation data were calculated for normally distributed parameters unless otherwise specified. Median and inter-quartile range (IQR) were calculated for parameters with skewed distribution. Continuous variables were evaluated using unpaired Student *t*-test and categorical data was evaluated with *χ*^2^ test with *p* < 0.05 considered statistically significant. Pearson’s correlation co-efficient was used to measure the degree of association between continuous data sets. Kaplan–Meier curves were used to demonstrate time free from mitral valve surgery with Log-rank test used to compare significance of stratified groups. Cox proportional hazards regression was performed to analyze time free from mitral valve surgery with adjustments made for age, sex, left atrial size (LA/Ao ratio), and left ventricular systolic function (fractional shortening).

## Results

**Fig. 2:**
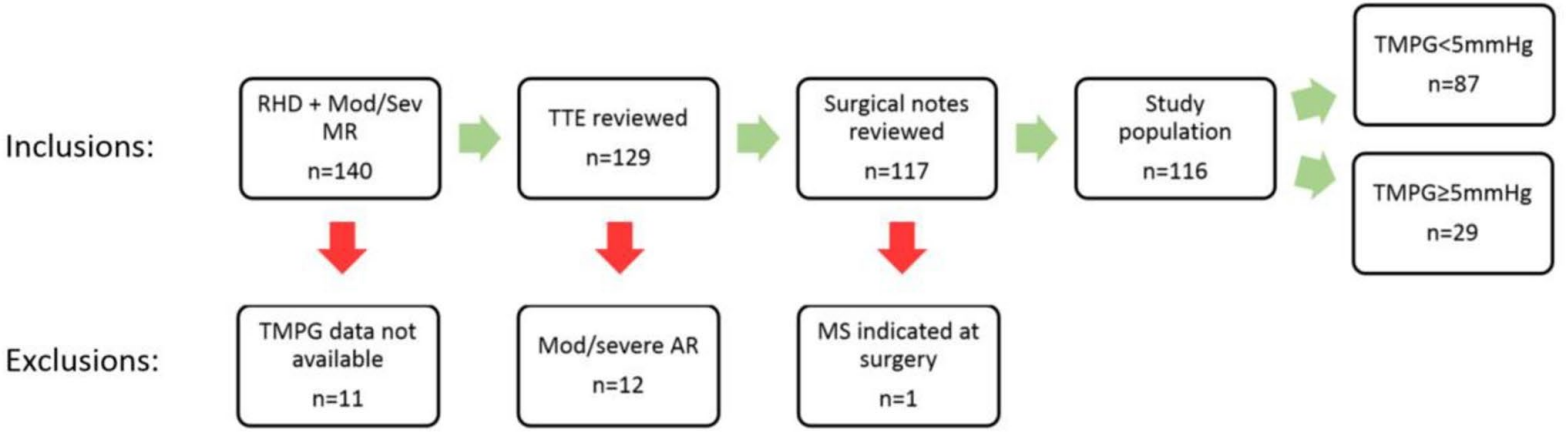
Derivation of study population and exclusions. The study cohort included children diagnosed with rheumatic heart disease who exhibited moderate or severe mitral regurgitation during the period of follow-up. Those with significant aortic regurgitation or mitral stenosis based on mitral valve area were excluded. *RHD* rheumatic heart disease; *MR* mitral regurgitation; *echo* transthoracic echocardiogram; *AR* aortic regurgitation; *MS* mitral stenosis

One hundred and forty patients met the initial inclusion criteria for echocardiographic analysis. Trans-mitral pressure gradient was obtained in 129 patients with 11 being excluded due to poor quality or unavailable images. A further 12 patients were excluded due to the presence of concomitant moderate or severe aortic regurgitation, and one was excluded due to significant mixed mitral valve disease leaving a final cohort of 116 patients (Fig. 2).

All participants were in sinus rhythm with median heart rate (HR) of 84 bpm (range 49–129 bpm) and all had moderate or severe MR determined through qualitative and semi-quantitative echocardiographic methods in accordance with the American Society of Echocardiography guidelines [13]. No patient had congenital heart defects detected. Patients were stratified into two groups based on the TMPG with one group comprised of those with elevated gradients (TMPG ≥ 5 mmHg) and the other included those with lower TMPG (< 5 mmHg). Table 1 provides a summary of the data with mean and range for the total population and following stratification by TMPG.

**Table 1:** Patient demographics and mitral valve features

Variable	TMPG < 5 mmHg (*n* = 87)	TMPG ≥ 5 mmHg (*n* = 29)	*p* value
Age at echo (years)	13 (2–23)	13 (6–22)	0.888
Female	49 (56%)	20 (69%)	0.230
Heart rate (bpm)	83 (49–129)	95 (67–129)	0.004
TMPG (mmHg)	2.3 (1–4)	6.4 (5–10)	< 0.001
MVA (cm^2^)	4.1 (2.0–7.2)	3.7 (2.1–6.0)	0.112
Aortic root dimension (cm)	2.48 (1.5–3.2)	2.48 (1.9–3.4)	0.938
Left atrial diameter (cm)	3.37 (2.2–5.4)	4.04 (2.8–5.3)	< 0.001
LA/Ao ratio	1.38 (0.93–2.06)	1.66 (1.12–2.32)	< 0.001
MV surgery	10 (11%)	18 (62%)	< 0.001
Fractional shortening % (*n* = 114)	34.6 (21–50)	36.8 (25–47)	0.083
Age at surgery, (years)	12 (5–18)	13 (7–21)	0.354
Time from echocardiogram to surgery (months)	9.8 (1–23)	11.2 (0–61)	0.746
Time from RHD to surgery (months)	23.7 (3–51)	25.6 (0–83)	0.840

Values are expressed as mean (range). P values represent differences between those with TMPG < 5 mmHg and TMPG ≥ 5 mmHg

*bpm* beats per minute; *TMPG* trans-mitral pressure gradient; *MVA* mitral valve area; *LA* left atrium; *Ao* aorta, *RHD* rheumatic heart disease

### Proportion Free from Mitral Valve Surgery

**Fig. 3:**
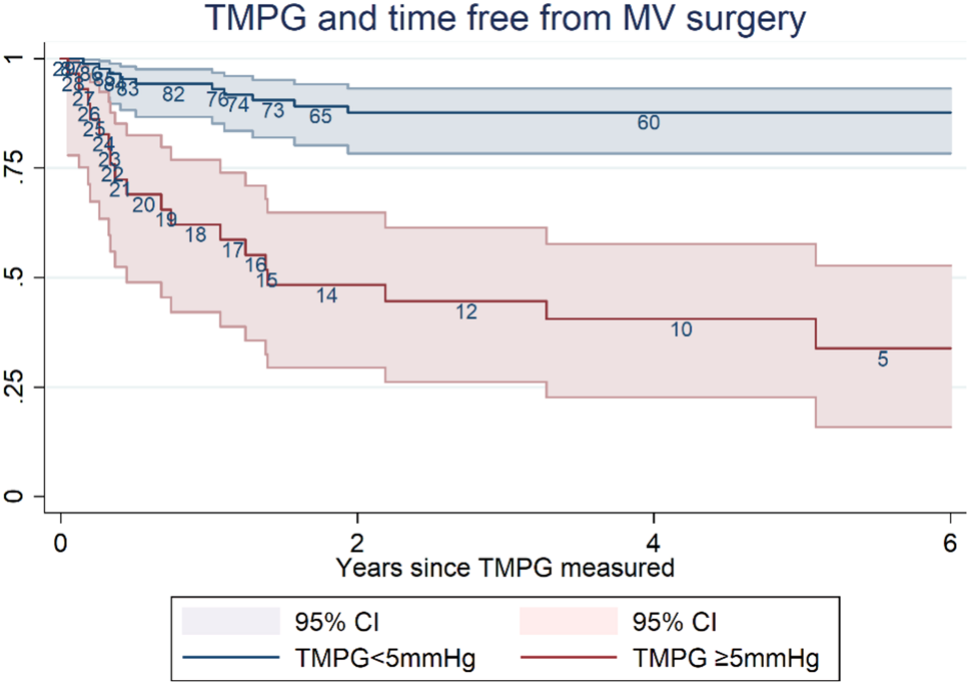
Increased risk of mitral valve surgery with elevated trans-mitral pressure gradient in those without significant MS. Children and young adults with moderate/severe rheumatic mitral regurgitation have increased rates of mitral valve surgery when TMPG is ≥ 5 mmHg. The blue line in the survival curve demonstrates the rate of mitral valve surgery in those with moderate/severe MR and TMPG < 5 mmHg. The red line shows the rate of surgery in those with moderate/severe MR and TMPG ≥ 5 mmHg. Hazard ratio 5.8 (CI 2.5, 13.5) adjusted for age, sex, LA size, & LV function for those with elevated TMPG

The incidence of mitral valve surgical events was highest within the first 18-month post-echocardiographic measurement of TMPG with 25 of 28 events occurring within this period, (Fig. 3). Of those with TMPG < 5 mmHg, the proportion free from surgery at 1 year was 0.94 (95% CI 0.86–0.97), compared to 0.62 (95% CI 0.42–0.77) in the ≥ 5 mmHg group. At 5 years, the proportion free from surgery was 0.88 (95% CI 0.78–0.93) in the < 5 mmHg group compared to 0.40 (95% CI 0.22–0.58). Population size diminished substantially in the ≥ 5 mmHg group beyond the first 2 years with most of the group having undergone mitral valve surgery.

### Mitral Valve Gradient ≥ 5 mmHg

Twenty-nine children (25%) had TMPG ≥ 5 mmHg (mean 6.5 ± 1.6 mmHg) with corresponding mean MVA 3.7 ± 1.0 cm^2^. Eighteen (62%) had mitral valve surgery during the follow-up period with a median time to surgery of 4.5-month (IQR 3.0–13.5 months) post-echocardiographic findings and a median time of 9.5-month (IQR 3.0–52.5) post-RHD diagnosis. Average age at the time of surgery was 13.1 years (range 7–21 years). Median heart rate was 95 bpm. Fractional shortening in this group ranged from 25 to 47% and mean 36.8%.

Elevated TMPG provides a significant risk of mitral valve surgery compared to TMPG < 5 mmHg with a hazard ratio of 5.8 (CI 2.5–13.5), *p* < 0.001 when adjusted for patient age, sex, left atrial size (LA/Ao ratio dichotomized as < or ≥ 1.5), and left ventricular systolic function (FS%) dichotomized as < or ≥ 40%.

### Mitral Valve Gradient < 5 mmHg

Eighty-seven children (75%) had TMPG < 5 mmHg (mean 2.3 ± 1.1 mmHg) with corresponding MVA mean of 4.1 ± 1.0 cm^2^. Ten (11%) required mitral valve surgery a median time of 9 months (IQR 3.5–14.5 months) post-echocardiographic findings and a median time of 19.5 months (IQR 6–41.75 months) post-RHD diagnosis. Mean age at the time of surgery was 11.5 years (range 5–18 years) and did not vary significantly from the ≥ 5 mmHg group (*p* = 0.460). Median HR was 85 bpm and was significantly lower than the ≥ 5 mmHg group (*p* = 0.004).

Fractional shortening ranged from 21 to 50% (mean 34.6%) and did not vary significantly from the ≥ 5 mmHg group (*p* = 0.067).

### Mitral Valve Surgical Events

All children undergoing surgery (*n* = 28) had severe MR and met the recommendations for intervention outlined in internationally endorsed guidelines including LV dilatation and/or onset of heart failure symptoms [12]. It is important to note, surgical indications were related to the severity of MR, *not* the presence of significant MS. The median TMPG for this group was 5 mmHg (IQR 3.3–7.8 mmHg). There were 15 children with severe MR who did not require surgery and this group had a median TMPG of 4 mmHg (IQR 2.5–4.0 mmHg) which was significantly lower than the group undergoing surgery (*p* = 0.011).

**Fig. 4:**
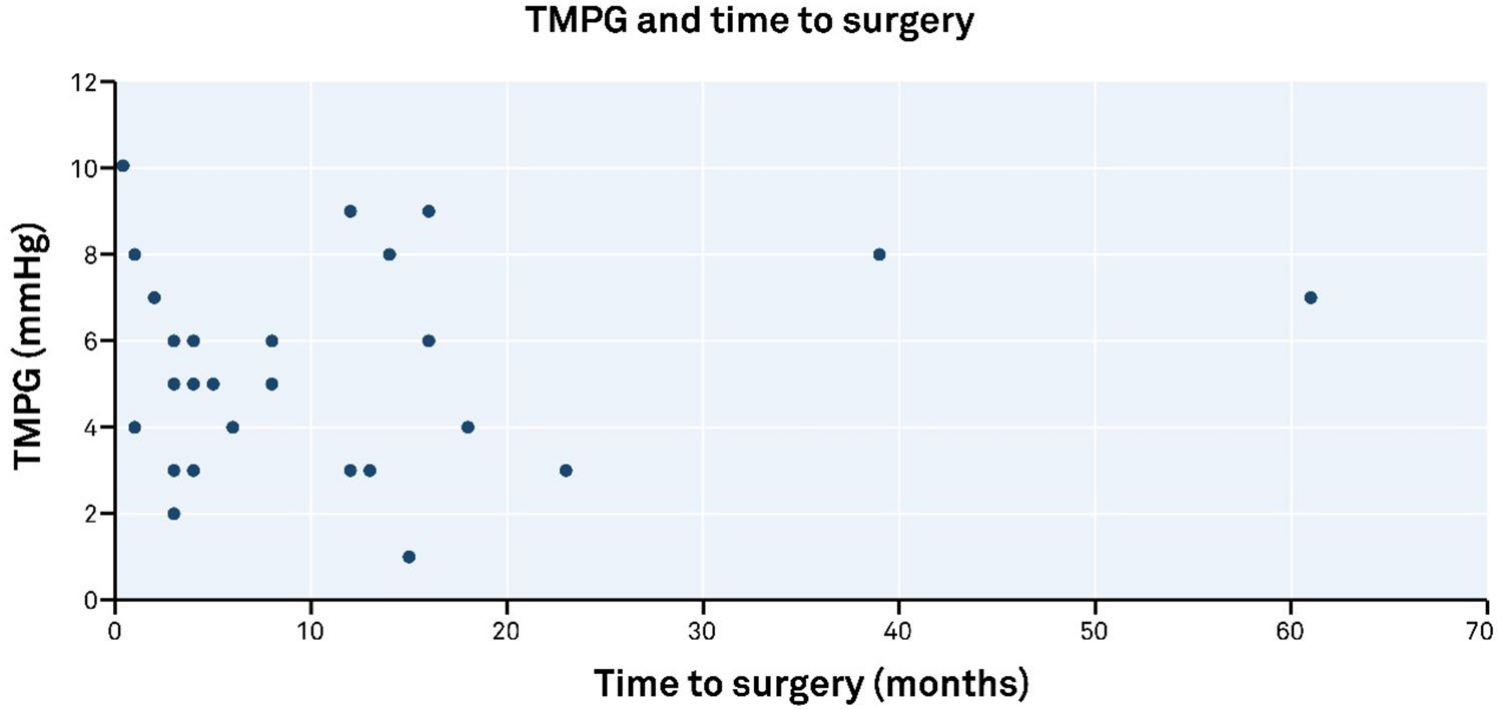
Trans-mitral pressure gradient (TMPG) and time to surgery. The severity of TMPG did not correlate with the time to mitral valve surgery with the majority of surgical interventions occurring with the first 18-month post-echocardiographic measurement of TMPG

The median time of follow-up for those who did not undergo MV surgery was 57 months for those in the TMPG ≥ 5 mmHg group (*n* = 11), and 49 months in the TMPG < 5 mmHg group (*n* = 77). There was no correlation between the TMPG severity and time to surgery (*r* = 0.092), (Fig. 4).

### LA/Ao Ratio

**Fig. 5:**
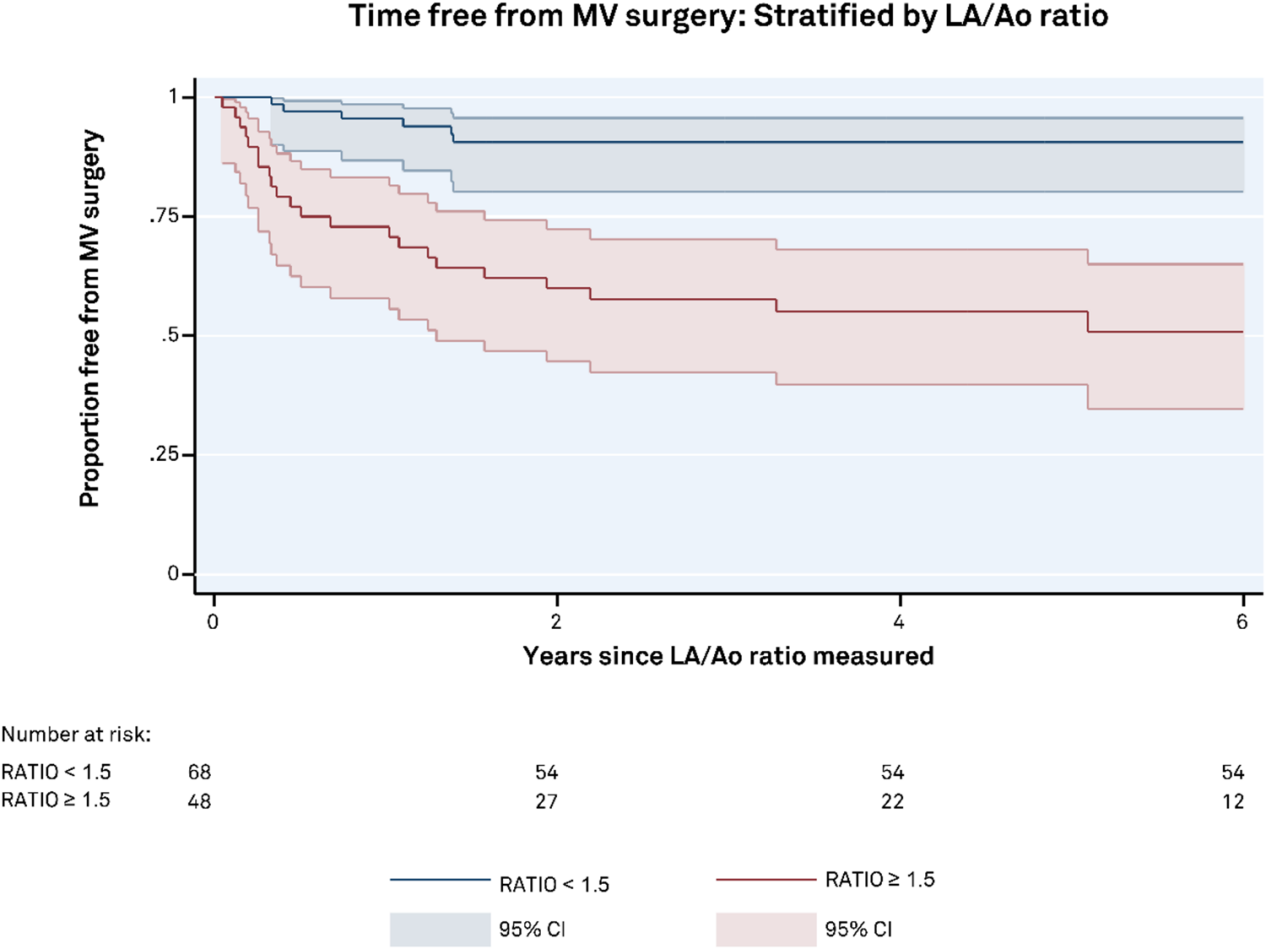
Time free from mitral valve surgery stratified by LA/Ao ratio. Left atrial size was a strong predictor of mitral valve surgery with a significantly higher number of patients requiring intervention in those with LA/Ao ratio ≥ 1.5

Stratification of data based on LA size shows a significant increase in the rate of MV surgery for those with LA/Ao ratio ≥ 1.5 (*p* < 0.0001). The survival curve shows 0.95 (CI 0.86–0.99) free from MV surgery at 1 year for the < 1.5 group, compared to 0.73 (CI 0.58–0.83) in those with LA/Ao ratio ≥ 1.5, (Fig. 5). At 5 years the < 1.5 group had 0.90 (CI 0.80–0.96) free from MV surgery, compared to 0.55 (CI 0.39–0.68) in the ≥ 1.5 group.

Increased LA size provides a significant risk of mitral valve surgery compared to LA/Ao ratio < 1.5 with a hazard ratio of 3.4 (CI 1.1–8.9), *p* < 0.027 when adjusted for patient age, sex, TMPG (dichotomized as < or ≥ 5 mmHg) and left ventricular systolic function (FS%) which was dichotomized as < or ≥ 40%, (Table 2).

**Table 2:** Comparison of LA/Aortic root ratio between TMPG groups with and without MV surgery

	TMPG < 5 mmHg	TMPG ≥ 5 mmHg	p value
**MV surgery**	n = 10	n = 18	0.644
LA/Ao ratio (cm)	1.66 ± 0.27	1.61 ± 0.29	
**Free from surgery**	n = 77	n = 11	0.008
LA/Ao ratio (cm)	1.34 ± 0.25	1.56 ± 0.25	
**p value**	< 0.001	0.162	

LA/Ao expressed as mean and standard deviation

*TMPG* trans-mitral pressure gradient; *MV* mitral valve; *LA* left atrium; *Ao* aorta

**Fig. 6:**
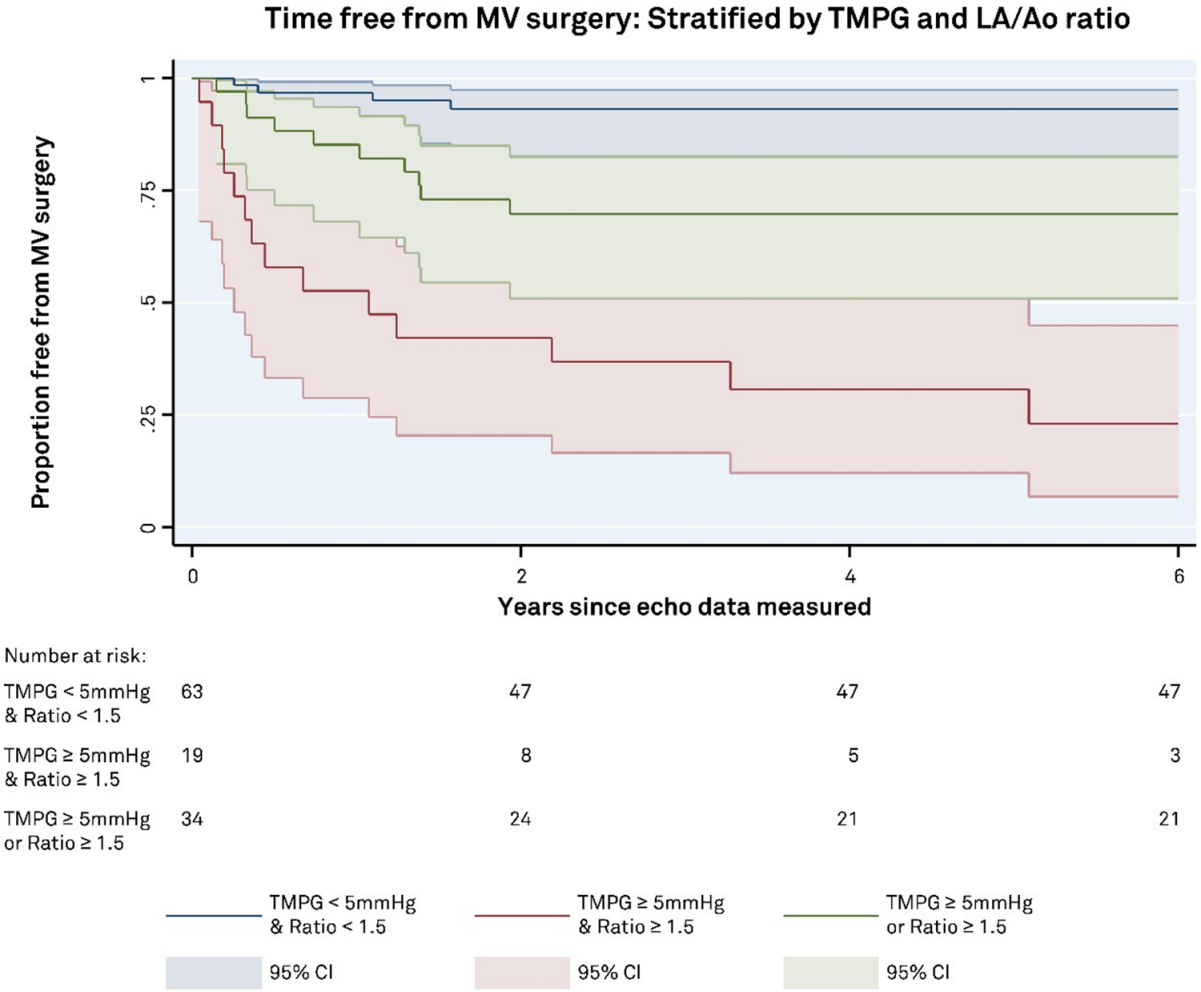
Survival curves stratified by trans-mitral pressure gradient (TMPG) and left atrial size. Stratification of patients by both TMPG and LA/Ao ratio reveals significantly higher rates of mitral valve surgery in those with elevated TMPG and left atrial dilatation. Those with TMPG < 5 mmHg and smaller left atrial size experienced lower rates of mitral valve surgery, while those with one factor (increased TMPG **or** left atrial dilatation) experienced intermediate rates of surgery

Trans-mitral pressure gradient and LA/Ao ratio are both independent predictors of MV surgery. Combining both parameters, three groups were examined: patients with both elevated TPMG *and* increased LA size (2 factors), patients with either elevated TMPG *or* increased LA size (1 factor), or lower TPMG *and* smaller LA size (0 factors). The group with TMPG < 5 mmHg and LA/Ao ratio < 1.5 have the least rates of surgery, while those with TMPG ≥ 5 mmHg and LA/Ao ratio of ≥ 1.5 have the highest rates and those with only one factor fell between the two (*p* < 0.0001), (Fig. 6).

## Discussion

### Elevated TMPG Linked to Mitral Valve Surgery

Consideration of TMPG in cases of significant MR offers a novel approach to the determination of the hemodynamic impact of this lesion and may reflect the increased loading conditions and changes in left atrial compliance that lead to heart failure in children [14, 15]. It also provides a useful prognostic target for monitoring children living with RHD. A higher proportion of children and young adults with increased TMPG required mitral valve surgery compared with those displaying gradients < 5 mmHg, despite the absence of significant MS in both groups. Importantly, only 62% of children were free from MV surgery at 1 year compared to 94% of the TMPG < 5 mmHg group. Elevation of TMPG in those with significant MR is well documented; however to our knowledge, this is the first time a link between TPMG and valve surgery has been documented in patients with rheumatic MR in the absence of significant MS.

### Left Atrial Dilatation Also Predicts MV Surgery

Echocardiographic parameters commonly used to evaluate the impact of significant MR include LA and LV size and pulmonary artery (PA) pressure. Chronic severe MR typically leads to increased chamber dimensions and elevated pulmonary arterial pressure; however, only changes in left ventricular size and function are considered indications for surgery with the presence of pulmonary hypertension being absorbed into the presence of heart failure symptoms [6]. The LA/Ao ratio, commonly used in pediatric cardiology, has been superseded by more reproducible and accurate estimate of LA size in adults, but is useful in children as it provides and indexed measure of left atrial size over a child’s lifespan. By comparing the LA to the aortic size, which is unaffected by MR volume overload, we get an estimate of proportional LA dilatation and are able to detect increased LA volume [9, 16]. The dilatation of LA reflected in the LA/Ao ratio mimics the survival curve of TMPG when using a cut-off ratio of 1.5, with 73% of MV surgical procedures in those with LA/A ratio ≥ 1.5 occurring within the first 12 months following echocardiographic data collection. Left atrial dilatation is expected in the presence of significant chronic MR and in those with both LA dilatation and elevated TMPG the highest number of MV surgical events occurred, suggesting further risk when both factors are present.

Our data also show that those undergoing future surgery had significantly increased LA dimensions at baseline compared to those who did not require surgery and in those who did not require surgery, the TMPG ≥ 5 mmHg group had a significantly larger LA than the TMPG < 5 mmHg group, despite both groups having significant MR.

### Possible Mechanisms for Elevation of TMPG

The pathophysiological impacts of significant valve disease are complex, resulting in chamber remodeling and changes in compliance [17, 18]; however, the total effect of these changes may be simplified by observation of the TMPG which is the product of the individual hemodynamic components. Schwammenthal et al. provided a link between atrioventricular (the incorporation of both left atrial and left ventricular) compliance and poor clinical outcomes, including elevation in pulmonary artery pressure in those with mitral stenosis [19]. While the etiology of MR is different to stenosis, the effect on LA compliance may be the same as is reflected by the elevation of TMPG in those reaching our surgical end-point. If this were the case, the relative contribution of elevated TMPG in those with mixed mitral valve disease may be irrelevant in the context of symptom development. Etiology is of course, very relevant for those considering surgical intervention.

Braunwald and Awe described a series of 10 patients some sixty years ago who presented with isolated chronic severe MR and dilated LA yet normal LA pressure determined through cardiac catheterization [20]. They hypothesized that chronic dilatation of the left atrium in some patients may result in an increase in compliance, permitting a normalization of atrial and pulmonary vascular pressures, yet a persistence of heart failure symptoms. Our data revealed 10 patients with TMPG < 5 mmHg who required mitral valve surgery. Eight of those registered LA/Ao ratios > 1.5. It is possible that LA remodeling and a shift in the pressure– volume relationship of the atrium as Braunwald suspected, is responsible for this finding; however, it is also possible that the reduction in TMPG is due to a decline in left atrial function [18] which we were not able to directly measure in this study. The advent of LA strain may be a useful advance in further understanding the precise mechanism of elevated TMPG in patients with significant MR.

### Effect of Heart Rate on TMPG

The velocity time integral (VTI) represents the volume of blood passing through an orifice, such as the mitral valve. If the volume of blood remains the same but the duration of flow (diastolic period) is reduced, the velocity must increase to maintain the stroke volume, thereby increasing the VTI and TMPG. Tachycardia results in a shortening of the diastolic filling period which may cause an increase in TMPG. Our study showed an average HR of 95 bpm for those with TMPG ≥ 5 mmHg, compared to 83 bpm for those with TMPG < 5 mmHg. This increase in HR may contribute to the increase in TMPG.

### Patient Management

Management pathways for children with severe MR are largely based on adult guidelines. Medical management of adults with moderate to severe chronic MR includes the use of diuretics, angiotensin-converting enzyme inhibitors, and occasionally B-blockers. Sampaio et al. demonstrated a reduction in LA and LV size along with improvements in MR severity (including rheumatic MR and leaflet prolapse) with the use of Enalapril [21]. Surgical intervention is recommended for symptomatic patients with chronic, severe MR and LV dilatation, and/or LV systolic dysfunction (Class I) and in asymptomatic patients with preserved LV systolic function but progressive increase in LV dimension (Class IIa) [6].

Mitral valve surgery carries a risk of late mortality in children with RHD with survival of 83% reported at 15 years [22]. Mitral valve repair is preferred in children, offering improved survival and freedom from reoperation compared with valve replacement [23, 24]; however, avoidance of surgery or at least delay until adulthood would likely be the preferred option provided symptomatic relief can be achieved. The rate of reoperation at 10 years following mitral valve repair in children ranges from 18 to 47% [22–24]. Surgical intervention may be an option for children in resource-rich countries; however, conservative management may be the only option for many living in other limited resource and high-risk regions, and detection of an elevated TMPG in those with significant MR may be a signal to provide more aggressive heart failure therapy if intervention or early mortality is to be avoided.

Timing of mitral valve surgery relies on echocardiographic features of severe disease, along with clinical signs and symptoms of heart failure [25]. While an increase in TMPG is expected in cases of significant MR, the gradient is not traditionally used to evaluate MR severity or as a predictor of outcomes, such as the need for surgical intervention [3, 8]. Based on the 2D planimetry of MVA, no patient in our cohort had significant MS at the time of echocardiography nor was MS recorded on the operation report for those undergoing mitral valve surgery, confirming that significant MR was the cause of elevated TMPG rather than stenosis.

### TMPG as a Prognostic Tool

The use of TMPG as a prognostic tool has not been investigated widely in cases of significant rheumatic MR in children. Bertrand, et al. have investigated the utility of TMPG in elderly patients with mitral annular calcification (MAC) and stratified his group based on TMPG and severity of co-existing MR [26]. Trans-mitral pressure gradient demonstrated an independent association with mortality, and MR was associated with mortality at low TMPG but not with high gradients. As Bertrand discusses, the presence of elevated TMPG is prognostic in the elderly, regardless of whether the mechanism for elevated gradient is MR or MS, and this is supported by the findings of our study. Differences may exist between our groups with our young cohort likely to have more vigorous and compliant LA and LV function compared to an elderly population with MAC; however, these parameters were not evaluated directly in either study.

It has been demonstrated previously in an adult cohort that MR regression is associated with improvement of loading conditions [27]. In adult patients, post-mitral valve repair (via surgical or trans-catheter techniques), an elevated TMPG predicts higher B-type natriuretic peptide levels and increased hospitalization and deterioration in NYHA functional class [28–30]; however, these findings may be attributed to iatrogenic stenosis post repair, rather than the presence of significant residual MR.

The acquisition of TMPG during echocardiography requires a degree of technical skill and experience however can be routinely measured in all children with satisfactory echocardiographic windows.

We have identified those at risk of early deterioration in the months following detection of elevated TMPG in the setting of significant MR, which appears to have relevance beyond the diagnosis and classification of MS. This group may benefit from increased monitoring and therapeutic optimization particularly in the first 18 months following detection of elevated TMPG.

## Limitations

Our observational study collected data from retrospectively captured echocardiographic images. Systolic blood pressure measurements at the time of MR assessment were not available thus preventing our ability to factor this into assessment of MR severity.

Due to the morphological mitral valve changes present in some patients and the lack of data verifying normal MVA in children, it is possible that some children had mild MS in addition to significant MR. Noting this, the smallest valve area in our cohort was 2.0 cm^2^ in a 4-year-old child which likely represents mild stenosis at worst.

In the absence of suitable apical images, fractional shortening was used to assess LV function which is not the gold standard in adult patients but is common practice in pediatric echocardiography as children rarely possess regional wall motion abnormalities.

Current recommendations suggest LA size is best assessed via calculation of biplane LA volume^31^; however, a lack of optimized apical views and the absence of height and weight data in many cases prevented our ability to assess chamber dimensions following contemporary guidelines. Nonetheless, the data used in this analysis represent a real-world example of RHD assessment in the remote environment of the Northern Territory of Australia which is serviced by visiting cardiology specialists.

This was a relatively small sample from a unique group of Aboriginal Australians with RHD living in the Northern Territory of Australia. It is uncertain whether our findings could be extrapolated to include children or adults with significant MR from other causes.

## Conclusion

Our data suggest that in children and young people with significant rheumatic MR, an elevated mean TMPG (≥ 5 mmHg) in the absence of significant MS is prognostic for mitral valve surgery. Prognostication is improved with the addition of LA/Ao ratio ≥ 1.5 which reflects the presence of LA dilatation. Further research is required to determine whether mitral valve surgery may be delayed or avoided through careful clinical management in this group.

The inclusion of TMPG in the echocardiographic assessment of children and young adults with significant MR may enhance medical management decisions and assist in planning for surgical intervention. In addition to monitoring left ventricular size and pulmonary pressure, TMPG ≥ 5 mmHg may be an indication of the imminence of heart failure supporting the need for close monitoring of patients meeting this criterion.

Future studies should extend the use of TMPG measurement to adults with significant MR and those with MR from causes other than RHD. Additionally, further research into the relationship of elevated TMPG and the development of heart failure symptoms, specifically the measurement of left atrial strain, may assist our understanding of the mechanisms of heart failure in those with significant MR.

## Data Availability

Original data can be made available upon request.

## Abbreviations

ARF: Acute rheumatic fever
FS: Fractional shortening
LA: Left atrium
LV: Left ventricle
MR: Mitral regurgitation
MS: Mitral stenosis
MV: Mitral valve
MVA: Mitral valve area
RHD: Rheumatic heart disease
TMPG: Trans-mitral pressure gradient
